# Supplementary material for: Causal Relationship between Aging and Anorexia Nervosa: A White-Matter-Microstructure-Mediated Mendelian Randomization Analysis
Source: Biomedicines. 2024 Aug 16;12(8):1874. doi: 10.3390/biomedicines12081874 (PMC11351342; doi:10.3390/biomedicines12081874)
Supplement: Supplementary file 1 [file biomedicines-12-01874-s001.zip › Supplementary Figure/Figure S4/Singe SNP plot of MR analysis for TL to ubm-b-1779.pdf]

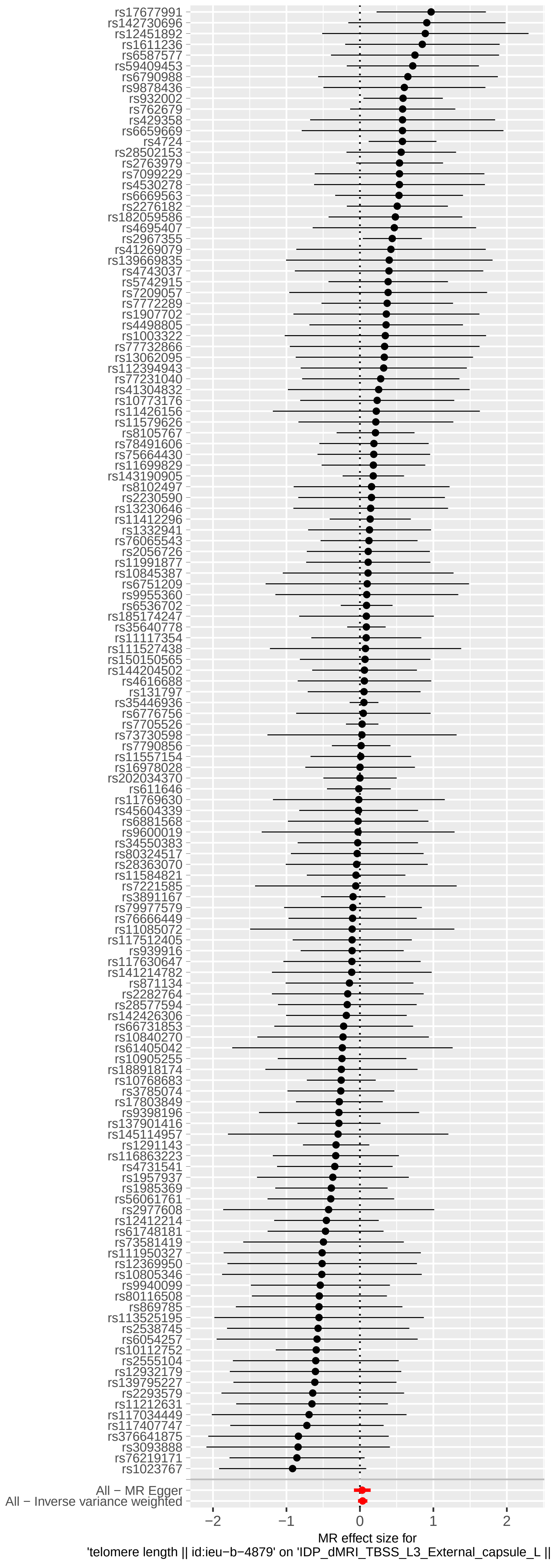
rs17677991
rs142730696
rs12451892
rs1611236
rs6587577
rs59409453
rs6790988
rs9878436
rs932002
rs762679
rs429358
rs6659669
rs4724
rs28502153
rs2763979
rs7099229
rs4530278
rs6669563
rs2276182
rs182059586
rs4695407
rs2967355
rs41269079
rs139669835
rs4743037
rs5742915
rs7209057
rs7772289
rs1907702
rs4498805
rs1003322
rs77732866
rs13062095
rs112394943
rs77231040
rs41304832
rs10773176
rs11426156
rs11579626
rs8105767
rs78491606
rs75664430
rs11699829
rs143190905
rs8102497
rs2230590
rs13230646
rs11412296
rs1332941
rs76065543
rs2056726
rs11991877
rs10845387
rs6751209
rs9955360
rs6536702
rs185174247
rs35640778
rs11117354
rs111527438
rs150150565
rs144204502
rs4616688
rs131797
rs35446936
rs6776756
rs7705526
rs73730598
rs7790856
rs11557154
rs16978028
rs202034370
rs611646
rs11769630
rs45604339
rs6881568
rs9600019
rs34550383
rs80324517
rs28363070
rs11584821
rs7221585
rs3891167
rs79977579
rs76666449
rs11085072
rs117512405
rs939916
rs117630647
rs141214782
rs871134
rs2282764
rs28577594
rs142426306
rs66731853
rs10840270
rs61405042
rs10905255
rs188918174
rs10768683
rs3785074
rs17803849
rs9398196
rs137901416
rs145114957
rs1291143
rs116863223
rs4731541
rs1957937
rs1985369
rs56061761
rs2977608
rs12412214
rs61748181
rs73581419
rs111950327
rs12369950
rs10805346
rs9940099
rs80116508
rs869785
rs113525195
rs2538745
rs6054257
rs10112752
rs2555104
rs12932179
rs139795227
rs2293579
rs11212631
rs117034449
rs117407747
rs376641875
rs3093888
rs76219171
rs1023767
All − MR Egger
All − Inverse variance weighted
−2
−1
0
1
2
MR effect size for
'telomere length || id:ieu-b-4879' on 'IDP_dMRI_TBSS_L3_External_capsule_L ||
